# The Impact of Rheumatoid Arthritis on Bone Loss: Links to Osteoporosis and Osteopenia

**DOI:** 10.7759/cureus.17519

**Published:** 2021-08-28

**Authors:** Roaa Kareem, Rinky A Botleroo, Renu Bhandari, Opemipo D Ogeyingbo, Rowan Ahmed, Mallika Gyawali, Nanditha Venkatesan, Abeer O Elshaikh

**Affiliations:** 1 Internal Medicine, California Institute of Behavioral Neurosciences & Psychology, Fairfield, USA; 2 Medicine, California Institute of Behavioral Neurosciences & Psychology, Fairfield, USA; 3 Internal Medicine, Manipal College of Medical Sciences, Kaski, NPL; 4 Public Health, Walden University, Minneapolis, USA; 5 Internal Medicine, Saint James School of Medicine, Park Ridge, USA; 6 Internal Medicine, All India Institute of Medical Sciences, Raipur, IND

**Keywords:** osteoporosis, fracture prevention, osteopenia, rheumatoid arthriitis, bone loss, prevention of osteoporosis

## Abstract

Rheumatoid arthritis (RA) is an autoimmune chronic connective tissue disease that produces persistent systemic inflammation, with joint inflammation leading to function loss and joint destruction. Low bone mass causes skeletal bone loss, commonly referred to as osteopenia or osteoporosis.

We conducted this literature review to examine the relationship between RA and osteoporosis and the variables contributing to this connection. We used articles from the US National Library of Medicine (PubMed), Google Scholar, Science Direct to access the required information. Eventually, our results concluded that RA could result in local periarticular and generalized bone loss. Many risk factors contribute to this association, such as chronic joints inflammation, glucocorticoid use, genetics, and estrogen hormone effects. Still, it is not clear yet whether this is due to a consequence of treatment, immobility, or the activity of the disease. There are many recommendations by the American College of Rheumatology for RA patients during the disease course to reduce the risk of osteoporosis development, which include early starts of disease-modifying anti-inflammatory drugs (DMARDs), doing a dual-energy x-ray (DXA) or quantitative ultrasound (QUS) for identifying a patient at risk of osteoporosis, taking vitamin D, calcium, and bisphosphonates. Further prospective studies and clinical trials are essential to provide a solid evidence-based recommendation that will help to prevent bone loss in RA patients.

## Introduction and background

Rheumatoid arthritis (RA) is a chronic disease that affects approximately 0.5-1% of the population [1,2]. It is most commonly autoimmune, showing up between ages 30 to 50; however, it can also happen at any age, and women are three times more prone to get RA than men [1]. Its exact etiology is unknown and both environmental and genetic factors can play a role [1]. Many patients have a human leukocyte antigen-DR4 serotype (HLA-DR4) association [1]. RA is an immunologically mediated event that can lead to joint injury by lymphocyte infiltration of synovium, synovial hyperplasia, activation of macrophage, lymphocytes, and fibroblast [1]. The disease manifests as symmetrical inflammatory polyarthritis of peripheral joints, mainly proximal interphalangeal (PIP) and metacarpophalangeal (MCP) joints, with synovial inflammation and proliferation [1]. The characteristic features of the disease are inflammation of synovial joints, accompanied by cartilage erosion and bone loss [1]. Associated with pain, swelling, tenderness, and morning stiffness, joint deformity may also develop due to persistent inflammation [1,2]. It causes significant morbidity, a reduced life span, and loss of work productivity.

Osteoporosis is a common disease of bone loss [1]. The reduced bone strength that predisposes to an increased risk for fractures in older individuals [1]. It can affect people from different ethnicity [1,3]. Many factors play a role in increasing the risk of developing osteoporosis, including age, postmenopausal state, glucocorticoid use, low body weight, low calcium, low vitamin D, immobility, and chronic inflammation [4-6]. 

We use T-scores for the diagnosis of osteoporosis in postmenopausal women and older men and, according to the WHO, the criteria for the diagnosis of osteoporosis is a T-score of -1 to -2.5 by bone mineral density (BMD) testing at the femur neck and lumbar spine [7]. In contrast, BMD values less than -2.5 are osteopenia [7].

This study aims to discover the association between RA and osteoporosis and highlight the risk factors that RA patients have that may cause or affect osteoporosis progress in the future. We used three databases: the US National Library of Medicine (PubMed), Google Scholar, and Science direct. The restrictions applied include: analyzing studies written only in English, free full text, humans subjects only, and adult patients.

## Review

Risk factors that increase the effect of RA on osteoporosis

Effect of Inflammation

**Figure 1:**
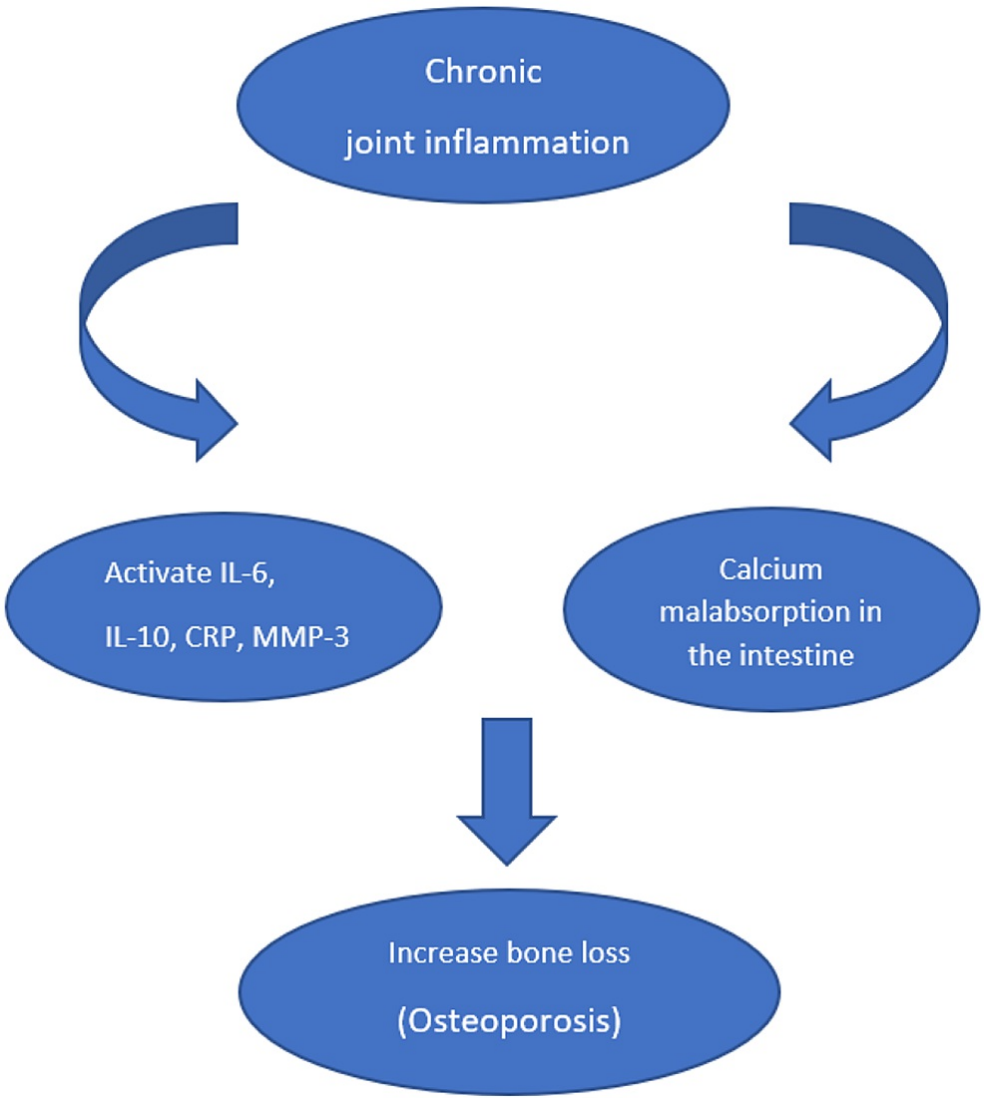
Effect of chronic joint inflammation on bone loss IL-6: interleukins 6; IL-10: interleukins 10; CRP: C reactive protein; MMP-3: Matrix metalloproteinase-3

In RA, the peripheral joints suffer from chronic inflammatory reactions [1]. This inflammatory reaction within the joint synovium leads to the production of multiple cytokines (tumor necrosis factor, interleukins-1, interleukin-6(IL-6)), resulting in activation of osteoclast that can mediate bone destructions [8]. Also, the inflammation in the joints increases bone absorption and makes the patient susceptible to bone loss and osteoporosis development [9]. In early RA, the periarticular osteoporosis development does reflect a disease activity because it is closely related to the acute phase reactant. However, later when osteoporosis is already established, it no longer reflects the disease activity [9]. A clinical study conducted by Yong W et al. selected 98 rheumatoid arthritis patients divided into an active stage group (56 patients) and a remission stage group (42 patients), compared the result with another 50 healthy people as a control group (the third group). The serum inflammatory factor changes were measured in the three groups and the result showed an increase in the level of IL-6, interleukins-10 (IL-10), matrix metalloproteinase-3 (MMP-3), and C reactive protein (CRP). All of these inflammatory factors are correlated positively with the disease activity of rheumatoid arthritis and osteoporosis [10]. A study conducted by Sambrook et al. showed impairment in calcium absorption in postmenopausal women with recent onset of rheumatoid arthritis compared to the control group [11]. It is possible that the disease inflammatory process is responsible for malabsorption of calcium or that the drugs taken by RA patients affect liver enzyme activity, which may, in turn, affect vitamin D metabolism in the liver and lead to bone loss and osteoporosis development [11]. Figure 1 given below illustrates the effect of chronic inflammation on the development of osteoporosis.

 *Effect of Medications on Bone Loss*

One of the primary drugs recommended for the treatment of RA is a corticosteroid, glucocorticoid (GC) [1]. Both systemic corticosteroid and intraarticular corticosteroid are proven to be risk factors for developing secondary osteoporosis and osteoporotic fracture [1,12]. The risk increases with the long duration of use and using a high dose [13]. An amount of more than 5mg daily and a treatment period of more than three months increase the risk of secondary osteoporosis highly [14]. It might also be possible that corticosteroids could cause osteoporosis, as a steroid known for its effect to suppress bone synthesis increases bone resorption and inhibits bone formation [13]. Many mechanisms play a role in the impact of steroids on bone remodeling, including increased sclerostin expression, upregulation of PPARγR2, increased receptor activator for nuclear factor-κB ligand RANKL, and soluble decoy receptor osteoprotegerin (RANKL/OPG) ratio, and decrease in the production of insulin-like growth factor-1 [13]. In one observational study published in 2017 by Ozen et al., 11,669 RA patients were recruited from rheumatologist clinics from 2003-2014. All followed with self-reported questionnaires semiannually during the two-nine years of follow-up period: osteoporosis was reported in 67.4% of these patients. While approximately 55% of the total patients reported using osteoporosis medication, the remaining patients never received treatment to reduce the risk of development of osteoporosis recommended by the American College of Rheumatology (ARC), which includes calcium/vitamin D supplementation, phosphonate, and regular fracture risk assessment for patients on GC [15]. A meta-analysis was done in 2014 by Siu et al. to study the impact of anti-rheumatic drugs on BMD in RA patients; the study recorded the change in BMD (ΔBMD) and standardized mean difference (SMD) of ΔBMD between the treated patients and the controls and calculated the 95%CI. The result shows steroid has a negative effect lumbar spine BMD, but not on hip BMD (SMD ΔBMD -0.30 [95% CI -0.55, -0.04], p = 0.02, I(2) = 52%), and there is significantly less bone loss on hand bone (SMD ΔBMD 0.51 (95% CI 0.20, 0.81), p = 0.001, I(2) = 0%) [16].

In a randomized, double-blind, placebo-controlled study by Laan et al. [17] to investigate the effect of low dose prednisone on bone loss for RA patients, all subjects received intramuscular gold salts and either prednisone 10 mg/d or placebo pills randomly, then tapered the dose between weeks 12 and 20. All patients had a follow-up period for additional 24 weeks. The BMD was measured for lumbar trabecular and a cortical region with quantitative CT and DXA at baseline and week 20 follow-up. The prednisone group had significant changes in BMD (9.5%; CI, 3.4% to 15.6%; p = 0.003), while the placebo-group patients have fewer changes in BMD (p > 0.2), which conclude to a marked vertebral bone loss in the initial months of prednisone treatment for RA patients, but this bone loss can be partially reversible as they noticed an increase in trabecular BMD between weeks 20 and 44 after discontinuation of prednisone (mean change, 5.3%; CI, 0.7% to 9.9%; p = 0.03).

In contrast to the known effect of corticosteroids on the increase of bone loss in RA patients and progressive osteoporosis development mentioned above [3], some studies showed that low dose prednisolone in early RA might counteract the negative impact of RA inflammation on bone tissue, especially in the hip and juxta-articular site [18]. However, low dose prednisolone didn’t prevent the systemic inflammatory consequences on the lumber bone; at the same time, this low dose prednisolone shows an increased risk of osteoporosis development, especially in postmenopausal women, due to the combined effect of steroid and postmenopausal state in suppressing bone synthesis [18]. In a randomized controlled trial by Haugeberg et al. to study the effect of intra-articular corticosteroid injections on bone loss in early RA, the patients were divided into two groups of 19 RA patients who received methotrexate alone and 21 RA patients who received intra-articular corticosteroid (IAST) injections with methotrexate (MTX) for three months into clinically inflamed joint. Then all patients received both MTX + IAST for the following nine months. The BMD for all patients was measured at baseline, three and 12 months, and the results of this study showed that the use of IAST injection combined with MTX for three months into inflamed joints has a protective effect on bone loss as it suppressed the inflammation of joints in the active RA [19]. This is in contrast to the long-term use of oral GCs, which can increase bone loss [15].

Effect of Genetics

One of the risk factors that can play a role in developing osteoporosis in RA patients includes genetics [1]. The process of bone remodeling is conformed by RANKL, nuclear factor-κB (RANK), and osteoprotegerin, which is an essential molecular coupling pathway between osteoclasts and osteoblast [20]. A cross-sectional study by Zavala-Cerna involved 82 women with RA, their mean age (50 ± 12) and RA duration (12 ± eight) years, and 52 healthy subjects. The genotype was measured for all subjects besides measuring the BMD at the femoral neck and lumbar spine. The study aimed to evaluate the association between C209T (rs3134069), T295G (rs3134070), SNPs C950T (rs2073617) in the TNFRSF11B (OPG) gene, and osteoporosis in RA. The result showed 46 (66.7%) had osteopenia/osteoporosis and 23 (33.3%) had normal BMD. RA patients had a higher prevalence of C allele for C950T SNP and, in contrast, both polymorphisms T245G and C209T did not reach statistical significance in allele distribution [20]. 

Xing-Hao Yu et al. conducted a series of genetic approaches by using studies from UK Biobank, which showed a complex genetic mechanism between osteoporosis and RA, and a shared genetic correlation reveals between estimated BMD and RA (p= 0.005). The pleiotropic analysis under composite null hypothesis (PLACO) analysis identified 74 pleomorphic loci (light novel) pleiotropic loci that mapped to 99 genes, which indicates the possible mechanism by which RA patients can get osteoporosis [21]. The same study showed an increase in genetic risk score (GRS) of the European RA population, which could lead to a decrease in estimated BMD (p = 3.77E-6, beta = −0.008) with a higher risk of fracture (OR = 1.012, p = 0.044). The Mendelian randomization (MR) showed that RA was causally associated with fracture risk (OR = 1.036, p = 0.004) and estimated BMD (beta = −0.021, p = 4.14E-05) [21]. In Asian patients, similar results were concluded, and the risk for osteoporosis causally increased by RA (OR = 1.130, p = 1.04E-03) [21].

Another study done by Mosaad et al. in Egypt revealed that the vitamin D receptor (VDR) gene polymorphisms (ApaI, TaqI, BsmI, and FokI) might play a role as a risk factor in RA patients for osteoporosis development; the study enrolled 128 RA patients with 150 control subjects and 30 postmenopausal osteoporotic females. The result revealed no significant associations for polymorphisms FokI and ApaI except for aa genotype (pc < 0.001). In contrast, a significant difference found in BsmI and TaqI (pc < 0.05) between RA and control group, rheumatoid factor (RF) titers were higher with genotypes (aa and bb), bone loss was high with Bb, C-reactive protein (CRP), and anti-citrullinated peptide (Anti-CCP) were more elevated with aa genotype. Also, the study showed that RA patients with osteoporosis have a higher Ff genotype than those without osteoporosis, which might be responsible for osteoporosis development in RA patients [22].

A study published in 2012 by Brambila-Tapia et al. investigated methylenetetrahydrofolate reductase (MTHFR) polymorphisms A1298C, C677T, and A163G in osteoprotegerin in RA; the scientists measured the BMD in 71 Mexican patients with RA at the lumbar spine and femoral neck and used the restriction fragment length polymorphism to analyze the genotyping for the three MTHFR polymorphisms (CC, CT, and TT). The result showed significant differences in the genotype frequencies of MTHFR C677T and reduced femoral neck BMD for the patient with osteopenia/osteoporosis; TT homozygotes had lower BMDs than CT genotype patients and a lower BMDs for TT and CT genotype patients than patients with the CC genotype. It was concluded that the associations of femoral neck BMD with the MTHFR C677T polymorphism and osteoporosis suggest that MTHFR C677T polymorphisms play the risk of developing osteoporosis in patients with rheumatoid arthritis [23].

Effect of Estrogen on Bone Loss

The female sex hormone, estrogen, plays a significant role in bone homeostasis and skeletal growth in both women and men [24]. The mechanisms for estrogen deficiency in bone loss are complex and multifaceted [24]. Bone cells (osteocytes, osteoblasts, and osteoclasts) express estrogen receptors (ERs) [24]. These are the same receptors expressed in bone marrow stromal cells, which are the precursor of osteoblasts and support for osteoclasts [24]. Estrogen has two signals available in bone cells, estrogen receptor alpha (Erα), and estrogen receptor beta (ERβ), but their distribution in the bone parts is not equal as (Erα) is predominant in cortical bone, which mediated most actions on bone cells, while ERβ is predominant in trabecular bone [24]. RA is a risk factor for early menopause, and the fact that RA patients reported experiencing late menarche leads to shorter exposure to estrogen, which is known to support skeleton growth and have a protective effect against the negative balance between bone resorption and formation [25]. The prevalence of osteoporosis in postmenopausal women can reach 50%, double the prevalence for non-RA women of the same age [25]. A study was undertaken by Sambrook et al. to examine the relationship of the sex hormones with osteoporosis in postmenopausal women with RA. They measured BMD and estrogen for 49 postmenopausal women with RA and compared them with 49 postmenopausal women without RA as a control group. The result of the study showed significant reduced femoral BMD (mean 0.72 gm/cm2 versus 0.80; p < 0.002), reduced dehydroepiandrosterone sulfate (DHEAS) (median 0.3 μmoles /liter versus 2.0; p < 0.001), and reduced levels of estrone (median 18 p moles /liter versus 49; p < 0.001). All might increase the risk of osteoporosis development in RA patients [26]. In 1993, Brink et al. conducted a double-blinded placebo-controlled comparative study to examine the benefit of adjuvant estrogen on BMD in RA patients. He enrolled 40 postmenopausal women with active RA, for 52 weeks and 33 patients completed the study. BMD was measured for all subjects before and after completion by DXA absorptiometry. The result for the estrogen group showed the concentration of sex hormones binging globulin increase, and the serum concentration of osteocalcin decreased and increased BMD for the femoral neck and lumbar vertebral spine; the result confirmed the positive effect of estrogen on bone density for postmenopausal women with active RA [27].

Prevention of osteoporosis in RA patients

Most studies showed that the use of anti-inflammatory treatment, including the disease-modifying anti rheumatoid drugs (DMARDs), reduce the rate of bone loss in RA patients [28,29]; Wijbrandts et al. conducted a prospective study about the effect of tumor necrosis factor (TNF)-alpha blocker (adalimumab) in RA patients. Fifty patients with active RA took adalimumab 40 mg subcutaneously for two weeks. They were allowed to take prednisone and MTX; at the same time, they measured the BMD at baseline, and one year after the treatment for the bone tested (the femur neck and lumbar spine). The study showed that TNF blockers might result in general bone loss arrest in contrast to the progressive bone loss that can happen after the use of the disease-modifying antirheumatic drug [30]. Another study done by Siu et al. showed significantly less bone loss in hand bones for RA patients treated with TNF inhibitors while no significant effect on BMD was observed on the hip and spine [16].

An observation study conducted at Oxford by Chen et al. investigates the BMD changes in RA patients who received biological target synthetic disease-modifying antirheumatic drugs (b/tsDMARD) over three years compared to patients with RA who received conventional synthetic DMARD (csDMARD); the study followed up 388 patients over three years and showed that long-term therapy with (b/tsDMARDs) may have protective effects on bone loss and patients who received concomitant anti-osteoporosis therapy and (b/tsDMARDs) therapy together experienced the greatest BMD preserving impact on bone loss [31].

In 2004, a prospective cohort study was conducted on the role of vitamin D by Merlino et al. for 29,368 women aged between 55-69 years without a history of RA at baseline. The subjects were followed over 11 years with measurement of vitamin D intake and the relative risk (RR) estimated for RA. The study showed the intake of Vitamin D was associated inversely with developing osteoporosis (RR 0.67; 95% CI 0.44, 1.00; p = 0.05) [32].

A study done by Hattori et al. enrolled 74 women with RA to explore the outcome of osteoporosis development in RA patients after two years of treatment with denosumab. They investigated baseline demographics and measured BMD for total hip (TH-) and lumbar spine (LS-) at baseline and at 24 months after treatment for each patient (-24m). Then, according to the percent change in BMD, they divided all cases into two groups: one-third of patients were in the non-good outcome group (LS-NG and TH-NG), and the other two-thirds of the patients were the good outcome group (LS-GO and TH-GO), the result showed (TH-BMD-24m) and (LS-BMD-24m) increased significantly from baseline. There was an increase in BMD% in LS (LS-BMD-24m) in the good group compared to LS in the non-good group, while the percentage of TH-BMD-24m showed no significant difference between groups, and the use of biologics in combination with denosumab was associated with a more substantial increase in total hip-BMD-24m. The result concluded the benefit of denosumab in increased BMD when used by RA patients with osteoporosis [33]. 

Bisphosphonates can prevent generalized bone loss by target osteoclasts [34]. The osteoclast is the main culprit of focal bone damage in inflammatory diseases like RA [34]. Bisphosphonates (alendronate) are used to treat osteoporosis [1]. A randomized controlled trial was done by Katayama et al. to investigate the effects of bisphosphonates on fracture incidence in 138 RA patients between 50-79 years. The subjects had been taking oral prednisolone for one year at a dose of 2-15mg/day combined with bisphosphonate therapy for ten months (risedronic acid 2.5mg/day or alendronic acid 5mg/day). X-rays were taken of the lumbar and thoracic spines at the start of bisphosphonate treatment and on completion of follow-up to measure the new vertebral fractures and compare between the two groups. The result showed that bisphosphonates have a strong prophylactic effect against fractures in RA patients [35]. The same study showed that alendronic acid has a stronger effect than risedronic acid for RA patients taking long-term corticosteroid therapy [35].

Additionally, not only are post-menopausal women with RA at high risk for osteoporosis development but even older men are at a high risk, according to a retrospective, cross-sectional study conducted by Kweon et al. in South Korea. The study followed 76 male RA patients aged over 50 years and compared to 75 healthy male individuals, by measuring BMA for left hip and lumbar spine (L1-4) with DXA. The result showed that men with RA have a 2.1 times higher risk for osteoporosis development compared with the healthy individuals (22.4% vs. 10.5%, p = .049). The study suggested that men aged over 50 years, especially those with low BMI and patients with higher RA disease activity, should get appropriate management of osteoporosis to prevent future bone loss [36].

**Figure 2:**
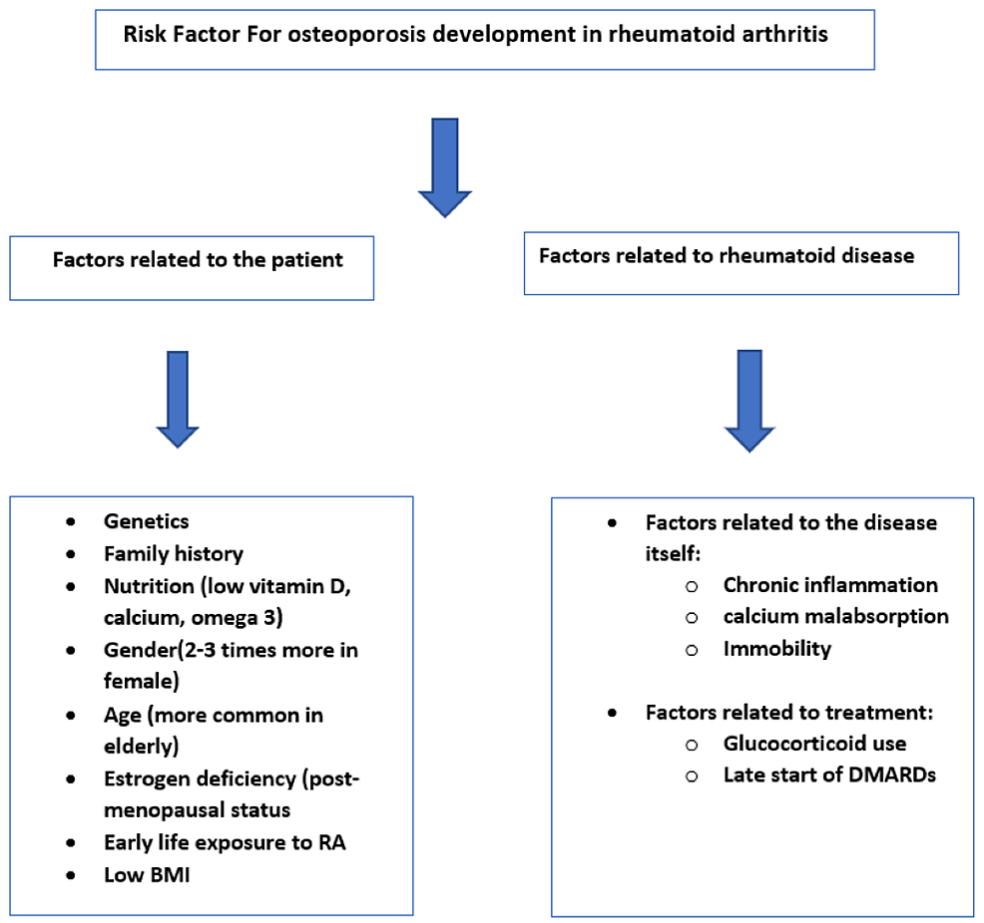
Risk factors for osteoporosis development in rheumatoid arthritis patients RA: rheumatoid arthritis; DMARDs: disease-modifying anti-rheumatology drugs

Until now, there are no anti-resorbing drugs that can arrest or change the progression of RA-related bone erosions, as erosions result from non-osteoclast effect mechanisms [3]. But we can decrease osteoporosis development and bone loss in RA patients by using anti-inflammatory treatment, including DMARDs, which could reduce joint inflammation and decrease the rate of bone loss in RA patients [8,29]. It is better to use corticosteroids for the short term and the lowest dose possible [29,12]. The ACR recommended increasing the awareness of RA patients about BMD and getting DXA or QUS done for identifying a patient at risk of osteoporosis [2,6,13]. Patients at risk should have additional focus and start taking vitamin D, calcium supplementation (particularly in GC users), anti-osteoporotic medications such as oral bisphosphonate or teriparatide (higher effect in reducing vertebral fracture risk), parenteral denosumab twice a year, and zoledronic acid once a year [4,13]. Figure 2 illustrates the risk factors for osteoporosis development in RA patients.

Limitations

Our literature review has few limitations; some of the selected studies were performed on a small sample population or only on one ethnicity, which impacted the study's power. Also, the study is limited to only three databases; the articles used to analyze the studies were in English language, free full text, humans only, and adult population patients may.

## Conclusions

We aimed to find the relationship between RA and osteoporosis and how they affect each other in this literature review. We found that most of our included studies showed RA is a vital risk factor for developing osteoporosis, and many factors can play a role in increasing this association including treatment RA patients with a high dose of glucocorticoid over a long duration, chronic joints inflammation, calcium malabsorption, age of the patients: post-menopausal women and older men above 50 years, genetics, and the estrogen hormone. These factors carry significantly elevated risks for developing osteoporosis and fractures in RA patients. To confirm these findings, we need well-designed, high-quality clinical studies in the future, to explore further the risk factors in RA patients leading to osteoporosis development with large sample sizes, different age groups, more studies to assess male patients with RA, in addition to well-designed clinical studies on the different anti-osteoporotic medications on the market such as bisphosphonates, denosumab, and odanacatib that can prevent osteoporosis, which can then help healthcare providers enlarge the possibilities of osteoporosis treatment and improve RA patient outcomes.
